# Synthesis of biowaste-derived carbon-dot-mediated silver nanoparticles and the evaluation of electrochemical properties for supercapacitor electrodes

**DOI:** 10.3762/bjnano.16.71

**Published:** 2025-06-24

**Authors:** Navya Kumari Tenkayala, Chandan Kumar Maity, Md Moniruzzaman, Subramani Devaraju

**Affiliations:** 1 Department of Chemistry, Vignan Foundation for Science, Technology and Research (Deemed to be University), Guntur, 522213, India; 2 Department of Chemistry, Mallareddy college of Engineering and Technology, Maisammaguda, Dhulapally, Secundrabad, Telangana, 500100, India; 3 Department of Chemistry, Gachon University, 1342 Seongnam‑daero, Sujeong‑gu, Seongnam‑si, Gyeonggi‑do 13120, Republic of Korea; 4 Department of Chemical and Biological Engineering, Gachon University, 1342 Seongnam-daero, Seongnam-si, Gyeonggi-do 13120, Republic of Korea

**Keywords:** asymmetric supercapacitor, carbon dots, fluorescence emission, green approach, silver nanoparticles

## Abstract

Herein, biowaste- (from *Pongammia pinnata* leaves) derived carbon dots (CDs) have been utilized as a mediator for the production of silver nanoparticles (PG-CDs-AgNPs) as a superior supercapacitor electrode. The methodology presented here is inexpensive and environmentally friendly as CDs play a role as capping, reducing, and stabilizing agent without addition of any chemicals. PG-CDs-AgNPs showed a particle size of 10 nm having excellent fluorescence emission in the blue region, and it has been explored as an electrode material for supercapacitor applications. The as-synthesized PG-CDs-AgNPs electrode exhibited the maximum specific capacitance of 540 F/g in a three-electrode study. The asymmetric supercapacitor (ASC) device with PG-CDs-AgNPs as the positive electrode reached the maximum specific capacitance of 200 F/g having a superior energy density of 71 W·h/kg at 1.5 A/g. Even at a high current density of 4 A/g, the ASC device reached a specific capacitance of 175 F/g, reinforcing its capability. The method described here provides a straightforward green approach towards biowaste-derived CD-mediated synthesis of AgNPs to produce efficient supercapacitor electrodes for energy storing.

## Introduction

The extensive usage of fossil fuels is a result from the rising demand for energy. However, the use of fossil fuels is not sufficient to attend the energy demand. Therefore, a notable research focus has been given to advancements in renewable energy storage systems. Secondary batteries and supercapacitors are promising alternatives for energy storage applications [1–2]. Through the electrostatic polarization of electrolyte solutions, supercapacitor devices store charge across the electric double layers [3]. Supercapacitors are highly sought-after energy storage devices, as their high power density, ultrastability, and rapid charge–discharge capability render them irreplaceable by traditional metal-ion batteries [4]. Typically, the functioning of supercapacitors involves two different kinds of charge-storing mechanisms: pseudocapacitance and electric double-layer capacitance (EDLC) [5]. The reversible redox processes are the primary charge-storage mechanism for pseudocapacitors [6]. Even though the capacitance value of pseudocapacitors is theoretically very high, it is often followed by poor stability and an inferior specific energy density. This is due to the poor conducting nature, slow redox kinetics, as well as structural collapse which occurs throughout the redox progression [7]. EDLCs are able to provide desired stability, superior power density, quick charge/discharge capacity, and an outstanding retention rate. Nanocarbons are the appropriate candidates for EDLCs due to their high surface charge-storage ability and high conductivity [8–10]. The development of electrode materials with an efficient EDLC and pseudocapacitor properties is essential [11–12]. The cost-effective production of efficient electrode materials for supercapacitors is also very important. Researchers are exploring a wide range of new and affordable electrode materials, while maintaining the superior power and energy density. In this work, we have synthesized cost-effective carbon-dot- (CD) mediated silver nanoparticles (AgNPs) for supercapacitor electrodes.

AgNPs have garnered substantial interest due to their capacity to effectively facilitate biological, optical, chemical, electrical, and industrial applications [13–14]. AgNPs are widely utilized in electrochemical energy storage applications given their exceptional chemical durability, high electronic conductivity, and surface chemical characteristics. Due to these characteristics, AgNPs possess the capacity to function as the electrode material for supercapacitors. Numerous reports have also shown that AgNPs actively improve the electrochemical characteristics of different electrode materials. Salve et al. reported a noteworthy charge-storing capacity of 367.16 mF/cm^2^ of the synthesized hybrid material, PGE/AgNPs/CS (pencil graphite electrodes/silver nanoparticles/chitosan) [15]. Lokhande et al. reached a specific capacitance (SC) of 424 F/g for the Kimchi cabbage extract mediated AgNPs [16]. The enhanced performance was credited to the fact that the AgNPs were smaller, which meant that there was a bigger surface area for operative conducting pathways. Nevertheless, chemical reduction of Ag is extremely hazardous and can be dangerous to humans and the environment [17]. Hence, safer nontoxic methods for the production of AgNPs are in high demand.

Green chemistry emphasizes the use of nontoxic chemicals and production pathways for environmental safety. Motivated by these approaches, researchers have synthesized AgNPs utilizing green synthetic strategies which incorporate the utilization of plant extracts as reducing agents and stabilizing agents instead of traditional synthetic reducing agents such as NaBH_4_ [16]. Eclipta alba, aloe vera, mint, and *Corchorous olitorious* are some of the plant extracts that have been selected as environmentally friendly reducing agents so far. However, the majority of these methods produce less stable and bigger particles, limiting their practical applicability and requiring more raw materials. CDs have a carbon core and surface functional groups which give them remarkable chemical and photophysical features, such as photostability, minimal toxicity, and tunable luminescence [18–19]. A significant number of studies has been carried out for developing CDs from inexpensive natural resources in many ways, which are also environment friendly. Instead of chemical precursors, plant materials such as mint, coriander, aloe, banana peel, and lemon were used to prepare carbon nanodots [20–24]. CDs have been recognized as green, effective agents for reducing metal ions to metal nanoparticles [25].

In this work, we focused on a green and facile synthetic way for the production of AgNPs utilizing CDs as reducing agent derived from *Pongamia pinnata* (*P. pinnata*) leaves. The novel aspect of this work is that a comprehensive green approach is verified throughout the process, starting with the choice of the CD precursor. The disadvantages of earlier research in this area include the need for a high reaction temperature and a drawn-out reaction process. However, this methodology does not involve any of these factors. The CDs have a dual behavior as it acts as a stabilizing and a reducing agent, resulting in the production of AgNPs with good water stability. The combination of EDLC-type CDs with pseudocapacitive AgNPs affords superior capacitive performance and stability, which are important for supercapacitor electrodes. The overall methodology along with the optical and electrochemical charge-storing performance have been schematically depicted in Supporting Information File 1, Scheme S1.

## Experimental

### Materials

All reagents of analytical grade were used for the experimental procedures. The details about all the chemicals are covered in Supporting Information File 1, section S1.

### Synthesis of carbon dots from *Pongamia pinnata* leaves (PG-CDs)

For the synthesis of PG-CDs, 100 g of *Pongamia pinnata* leaves powder was taken into 1 L of water and stirred for 15 min at 60 °C on a magnetic stirrer. Procedures for the preparation of *Pongamia pinnata* leaves powder are discussed in Supporting Information File 1, section S1. Then, it was filtered by a Whatman filter paper and the extract was collected and placed on a magnetic stirrer with a sand bath maintained at 120 °C for 24 h. The color of the solution changed from orange to brown confirming the reaction progresses. After that, it was left alone until the reaction solution reached room temperature. Centrifugation was conducted for 5 min at 3000 rpm, followed by syringe filtration (0.22 µM) to discard larger agglomerated particles. Finally, the clear brown solution of PG-CDs was then kept in a refrigerator for additional experiments.

### Synthesis of carbon-dot-mediated silver nanoparticles (PG-CDs-AgNPs)

The method for synthesizing AgNPs employing PG-CDs as both the reducing and stabilizing agents is as follows. PG-CDs-AgNPs were synthesized by adding the PG-CDs solution to 1 mM AgNO_3_ in 1:10 V/V ratio in 100 mL of distilled water. The reaction was kept at room temperature undisturbed. The color changing from brown to dark brownish black confirmed the synthesis of PG-CDs-AgNP within 2 h. After 2 h of reaction, the sample was collected for UV–vis studies. The UV–vis spectra were also implemented at 12 and 24 h, respectively. The growth and the formation of PG-CDs-AgNP were recorded by using UV–vis spectroscopy.

Supporting Information File 1, section S1 contains the details of the material characterization techniques.

## Results and Discussion

Plant extract-mediated production of nanoparticles from noble metal precursors are well reported. In the present work, waste *P. pinnata* leaf-derived CDs was utilized for the reduction of Ag^+^ to AgNPs. The efficient reduction of Ag(I) to Ag(0) at optimal concentrations of CDs confirmed the successful production of AgNPs from silver nitrate (AgNO_3_). The adding of a small amount of PG-CDs to a 0.1 mM AgNO_3_ solution (i.e, 1:9) produced AgNPs, as evidenced from the progressive color change of the solution as shown by surface plasmon resonance (SPR).

### Structural and morphological analysis

**Figure 1:**
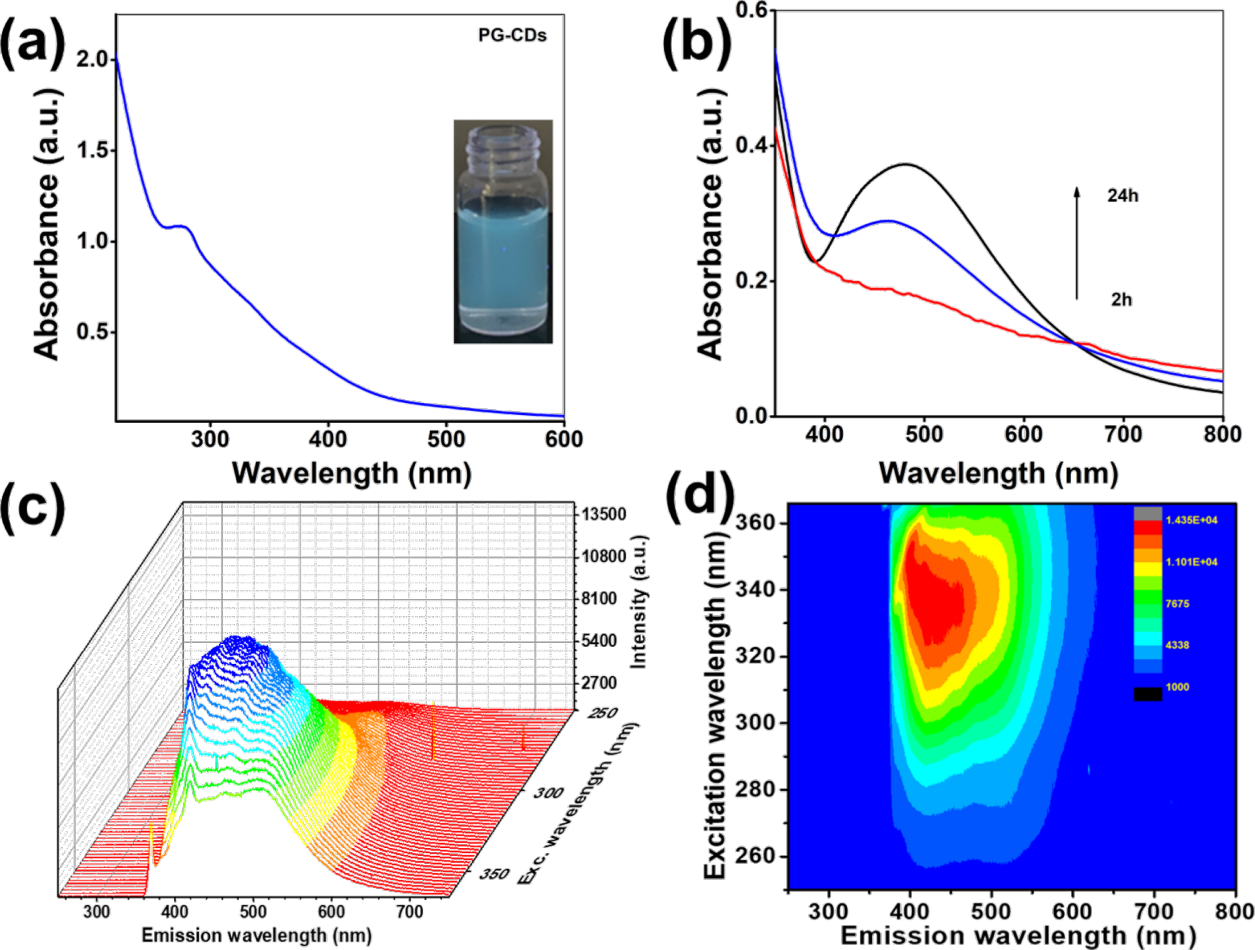
(a) UV–visible absorption spectrum of PG-CDs (the inset shows the digital photographic image of PG-CDs under the UV-illuminator). (b) UV–visible absorption spectra of PG-CDs-AgNPs at different time intervals. (c) Excitation–emission PL spectra of PG-CDs-AgNPs. (d) Excitation–emission contour map of PG-CDs-AgNPs.

The optical properties of PG-CDs and PG-CDs-AgNPs were studied using UV–visible spectroscopy and photoluminescence (PL) spectroscopy. An absorption peak at 275 nm was visualized in the UV–visible absorption spectrum of the PG-CDs, which is represented in Figure 1a. This peak might be accredited to the π–π* transition of conjugated (C_sp²_=C_sp²_) aromatic domain of the PG-CDs [24]. Under the ultraviolet illuminator (365 nm), the brownish/red aqueous solution of the PG-CDs exhibited intense blue fluorescence (inset in Figure 1a), indicating the successful formation and photoluminescent nature of CDs. The formation of PG-CDs-AgNPs in the nanoscale is established by UV–visible spectroscopy by the appearance of SPR peaks at 475 nm [26]. The gradual emergence of absorption bands of PG-CDs-AgNPs was acquired at various time intervals to study the production and growth of nanoparticles over time. The gradual growth of PG-CDs-AgNPs has been checked and depicted in Figure 1b. The SPR peak was noticed within 2 h of reaction with PG-CDs, with a visible change in color of the solution to brownish black. The gradual increase in the peak intensity without any spectral shift of absorption maxima (λ_max_) suggests that with increasing reaction time uniform-size stable nanoparticles were formed [27]. The excitation–wavelength-dependent emission spectra of the as-synthesized PG-CDs-AgNPs were recorded. As the excitation wavelength rises from 250 to 370 nm, the PL emission intensity gradually rises and the maximum PL emission intensity (430 nm) was attained at the excitation wavelength of 340 nm as displayed in Figure 1c. Figure 1d displays the excitation and emission contour map of PG-CDs-AgNPs, which suggests the PL emission in the blue range.

**Figure 2:**
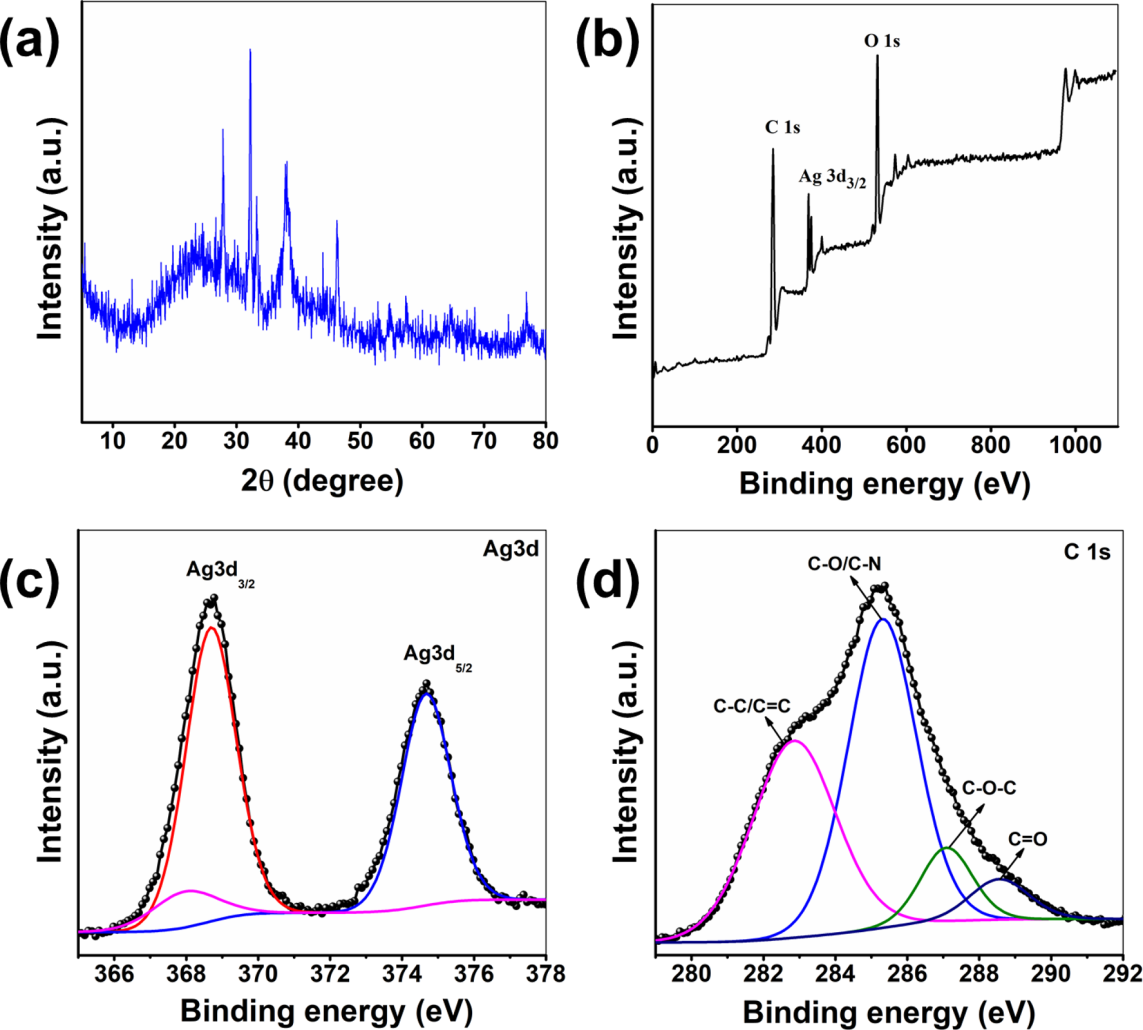
(a) XRD patterns of PG-CDs-AgNPs; XPS analysis of PG-CDs-AgNPs: (b) XPS survey profile; deconvoluted XPS spectra of (c) Ag 3d and (d) C 1s.

The crystalline nature of the PG-CDs-AgNPs was established from the X-ray diffraction pattern. Figure 2a displays an XRD pattern of the PG-CDs-AgNPs synthesized via PG-CDs mediated reduction. According to the XRD pattern, the phase composition could be classified according to the face-centered cubic structure of silver. The XRD spectrum showed that the formed PG-CDs-AgNPs were nanocrystals, as confirmed by the peaks at 2θ ≈ 28.20°, 32.66°, 38.61°, 46.60°, and 57.86°. These Bragg diffraction peaks are equivalent to the (110), (111), (121), (200), and (311) planes, which can be seen in the face-centered cubic structure of silver [28–29]. The XRD analysis of the PG-CDs-AgNPs produced via PG-CDs mediated reduction resembles the crystalline phase as referenced by (JCPDS File No. 84-0713) data [30].

**Figure 3:**
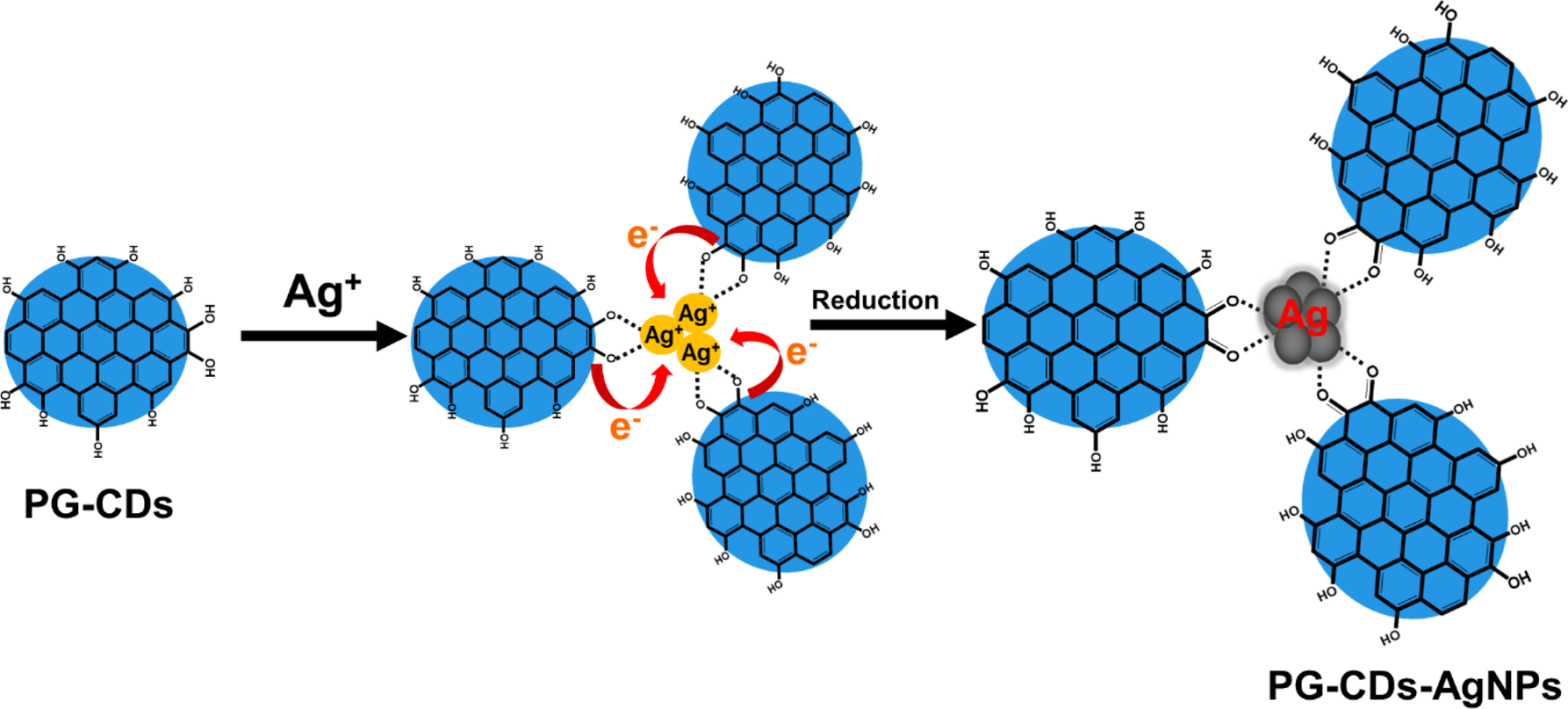
Formation and stabilization of PG-CDs-AgNPs.

Furthermore, the XPS analysis was implemented to study the elemental composition of PG-CDs-AgNPs and the corresponding chemical interaction between PG-CDs and AgNPs, as depicted in Figure 2b–d. As illustrated in Figure 2b, XPS total survey profile of the PG-CDs-AgNPs is composed of carbon and silver elements, with binding energies of 285 and 368 eV, respectively. The high-resolution individual elemental XPS spectra of C 1s and Ag 3d are exhibited in Figure 2c–d. As shown in Figure 2d, the C 1s spectrum of the PG-CDs-AgNPs composite exhibited four primary peaks at binding energies of 282.9, 285.3, 287.1, and 288.5 eV. These peaks could be ascribed to the C–C (sp^3^)/C=C (sp^2^), C–O, C=O, and O–C=O groups, respectively [31]. In addition, the Ag 3d spectra (Figure 2c) of PG-CDs-AgNPs exhibited two characteristic peaks at binding energies of 368.7 and 374.6 eV equivalent to Ag 3d_5/2_ and Ag 3d_3/2_, respectively [32]. It has been reported that the difference between the two peaks was around 6 eV, which indicates that silver was present in the PG-CDs-AgNPs in the form of nanoparticles of zero-valent silver [33]. Therefore, the XPS results proved that the PG-CDs-AgNPs were successfully synthesized. Additionally, CDs triggered the reduction of Ag^+^ to Ag(0) via oxygen-containing functionalities on their surfaces. The electrostatic attraction between the oxygen-containing functionalities on the surface of CDs binds with the positively charged Ag^+^ ions, which were then reduced by electron transfer from PG-CDs to the Ag^+^ ions [27]. The catechol moiety present in PG-CDs can form chelation with Ag^+^ ions. The Ag^+^ having a very high oxidation potential can easily be reduced to Ag(0) with concomitant oxidation of catechol to quinones. The quinones that are simultaneously created stabilize the AgNPs which are formed when the as-formed Ag(0) atoms in the reaction mixture collide with one another. This has been confirmed by the C 1s spectrum of CDs. The C–O group peak showed a decrease in intensity while the C=O group peak showed an increase in Figure 2d. This suggests that during the formation of the PG-CDs-AgNPs, the C–O groups on the surface of the CDs were oxidized and converted to C=O groups [34]. The mechanism for the production of PG-CDs-AgNPs has been illustrated in Figure 3.

The surface area of the supercapacitor electrode material is an important feature that can aid in surface charge storage. The specific surface area of the PG-CDs-AgNPs was determined using the Brunauer–Emmett–Teller (BET) analysis via the N_2_ adsorption–desorption method, and equivalent BET isotherms are depicted in Supporting Information File 1, Figure S1a. The surface area of PG-CDs-AgNPs was found to be 83.04 m^2^/g. A uniform nanoscale particle distribution of PG-CDs-AgNPs was responsible for the enhanced surface area [35]. The pore size distribution plot of PG-CDs-AgNPs suggests the mesoporous characteristics (Supporting Information File 1, Figure S1b), which, via the diffusion of electrolyte ions, is highly efficient for electrochemical charge storage.

**Figure 4:**
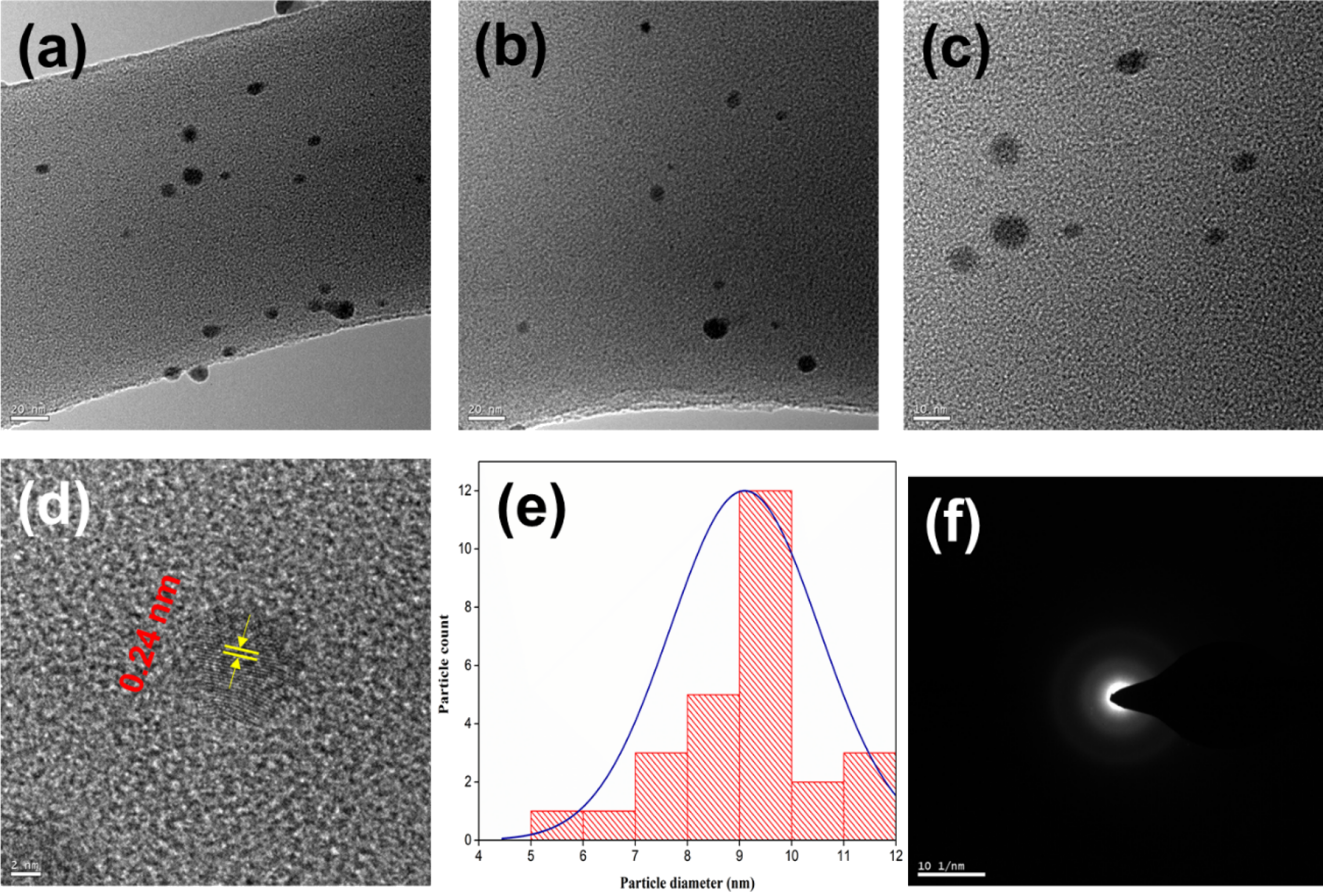
TEM images (a–c) of PG-CDs-AgNPs; (d) HRTEM image of PG-CDs-AgNPs, (e) average particle size distribution histogram, and (f) SEAD patterns PG-CDs-AgNPs.

The size distribution, morphology, and crystallinity of PG-CDs-AgNPs were further characterized by SEM and TEM analysis. The SEM image of PG-CDs-AgNPs (Supporting Information File 1, Figure S2a) reveals that PG-CDs-AgNPs possess spherical and distorted spherical structure. Supporting Information File 1, Figure S2b depicts the EDX mapping of PG-CDs-AgNPs, which proves the existence of Ag, C, and O in PG-CDs-AgNPs, and the atomic weight percentage (%) of the corresponding elements are given in the inset table (Supporting Information File 1, Figure S2b). The elemental mapping analysis for the PG-CDs-AgNPs proves the existence of O, C, and Ag (Supporting Information File 1, Figure S2c–e). Figure 4a–c displays TEM images of PG-CDs-AgNPs with different scale bars exhibiting that the particles are spherical and uniformly distributed. Figure 4d is the HR-TEM image of PG-CDs-AgNPs, exhibiting a well-resolved lattice spacing of 0.24 nm equivalent to (111) lattice planes of silver [36]. The clear visible lattice fringe indicates that the particles are crystalline. The average particle size distribution histogram depicted that the diameter of PG-CDs-AgNPs were approximately 9–10 nm, as depicted in Figure 4e. The narrowness of the average particle size distribution plot was well supported by the uniform particle size distribution shown in TEM images. Figure 4f is the selected area electron diffraction (SAED) pattern of PG-CDs-AgNPs, exhibiting a ring-like diffraction pattern indicating crystalline nature. The ring-like diffraction suggests the (111) plane. The symmetry in the diffraction spots indicates that the spherical particles possess a high degree of crystallinity. Thus, both the SAED pattern and HRTEM image suggest that the synthesized spherical PG-CDs-AgNPs are nanocrystals.

### Electrochemical properties of the synthesized electrode

#### Three-electrode analysis

Firstly, utilizing a three-electrode configuration in an aqueous electrolyte (1 M aqueous KCl), the electrochemical properties (capacitive characteristics, charge transportation, and charging/discharging) of PG-CDs-AgNPs were examined within a 0–0.8 V potential window. Aqueous (aq.) electrolytes are inexpensive, readily accessible, and environmentally benign. In this case, KCl (aq.) is neutral and contains small-sized, highly mobile K^+^ and Cl^−^ ions. These ions should be easier to reach in order to optimize the electrochemical activity, which can then optimize the capacitive response. Supporting Information File 1, section S1 contains the details of the electrochemical characterization techniques.

**Figure 5:**
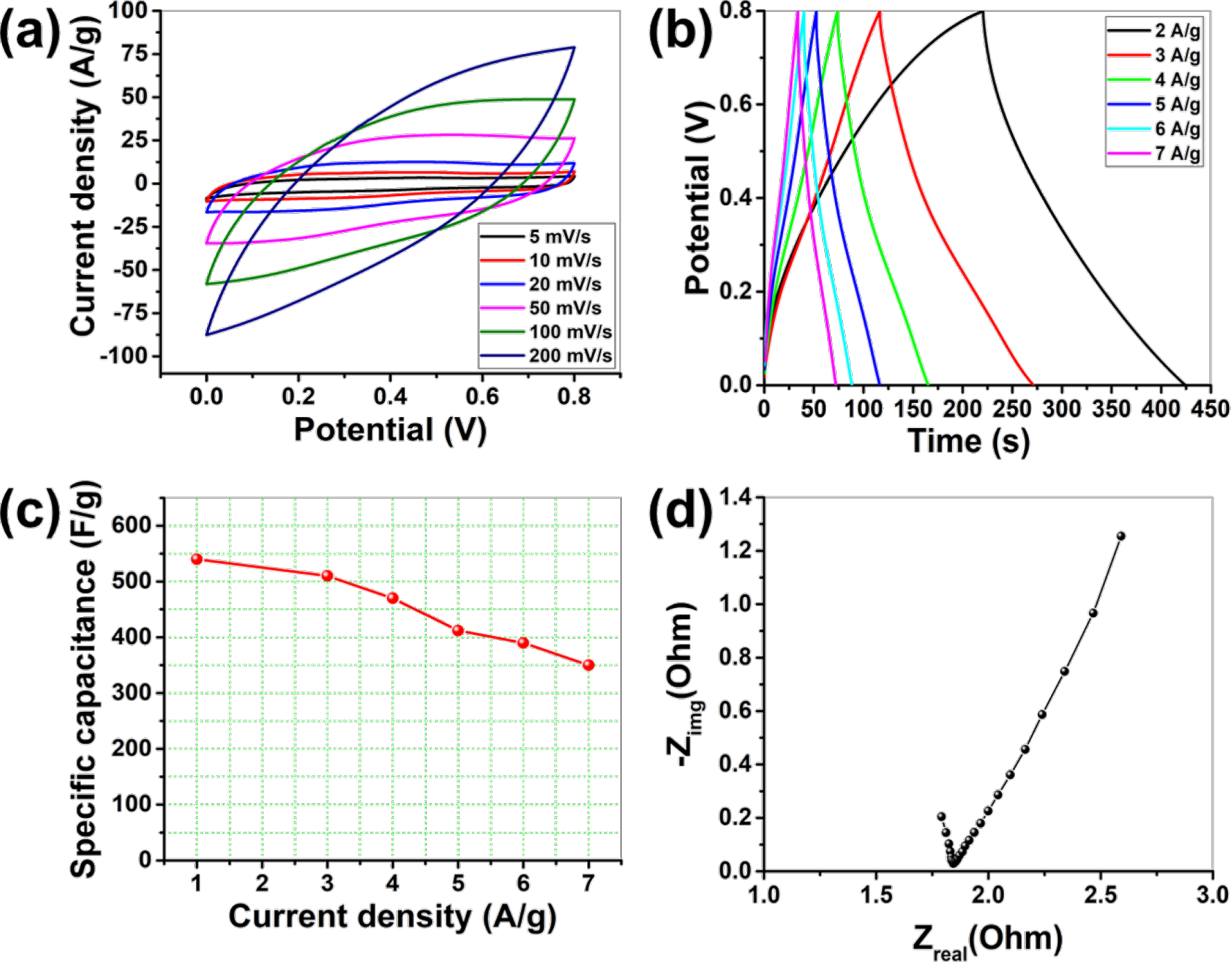
Electrochemical analysis of PG-CDs-AgNPs in 1 M aq. KCl using a three-electrode set-up: (a) CV plots of PG-CDs-AgNPs at various scan rates (5–200 mV/s), (b) GCD analysis of PG-CDs-AgNPs at various current densities, (c) SC as a function of current density of CDs-AgNPs electrode at various current densities, and (d) Nyquist graph of PG-CDs-AgNPs electrode.

Figure 5a depicts the CV plot of PG-CDs-AgNPs electrode at various scan rates (5–200 mV/s). It is evident through the PG-CDs-AgNPs CV graph that as the scan rates are increased, the current densities rise owing to the quicker diffusion kinetics on the electrode surface [37]. A lower scan rate means that the entire voltammogram takes longer to complete than a high scan rate. At low scan rates, diffusion layers extend further from the electrode surface, resulting in reduced electrode flux compared to that of higher scan rates. This occurs because current densities are influenced by the rate of scanning [37]. The higher current density at 200 mV/s suggests that, at this specific scan rate, the ion transport rate and the reactions for the interfacial charge storing are possible. The good capacitive characteristics and faster reversible electrochemical reactions of the electrode are also indicated by the comparable cyclic voltammogram at high scan speeds. Electric double-layer capacitance, not pseudocapacitance, is the main charge-storage mechanism for PG-CDs-AgNPs, as evidenced by the quasi-rectangular CV profile [38]. The electrochemistry of any electrode material is purposefully controlled by the composition of the electrolyte. Since KCl is used as a neutral electrolyte, PG-CDs-AgNPs exhibit capacitive behavior through a surface-charge-storing EDLC-type mechanism. Given the low charge density of K^+^ ions, which facilitates its access to the electrode during the electrochemical reaction, it may be concluded that this occurrence may have an impact on the performance of the electrochemical reaction. The deintercalation and intercalation of electrolyte ions on the surface of electrode, the efficient dispersion of electrolyte ions within the pores of PG-CDs-AgNPs and on their surface, and the high mobility of cations and anions within the electrolyte are the three mechanisms that allow PG-CDs-AgNPs to store charge during electrochemical reactions.

In order to ascertain the SC of PG-CDs-AgNPs, the galvanostatic charge/discharge (GCD) experiment was further implemented within the potential window of 0–0.8 V using the similar electrolyte and electrode configurations as the CV test (Figure 5b). The symmetric GCD profiles of PG-CDs-AgNPs demonstrate the neutral reversibility of the electrolyte and predominant EDLC behavior during the charge–discharge progression. Additionally, the GCD analysis showed that PG-CDs-AgNPs had 100% coulombic efficiency. Supporting Information File 1, Equation S1 was considered to measure the SC from the discharge time obtained from the GCD measurement. Higher SCs are demonstrated by electrode materials with longer discharge durations. At 2 A/g, the obtained SC for PG-CDs-AgNPs was found to be 540 F/g. Figure 5c exemplifies plots of the current density as a function of SC of PG-CDs-AgNPs at multiple current densities. Due to its superior rate capability at a high current density of 7 A/g, this electrode even had a decent SC of 350 F/g. Only around 35% of the initial SC is lost by the PG-CDs-AgNPs at a high current density. With respect to time, the electrolyte ions stimulated the electrode material, which was responsible for the decent rate capability and superior capacity of the PG-CDs-AgNPs electrode.

To evaluate the capability of charge carrying within the electrode and the capacitive property of PG-CDs-AgNPs, electrochemical impedance spectroscopy (EIS) measurements were also implemented. The Nyquist plots of the PG-CDs-AgNPs are displayed in Figure 5d. The charge-transfer resistance (*R*_ct_) in the high-frequency range is indicated by the semicircle diameter [39–40]. How facile electrons or ions can be moved throughout the electrode and electrolyte–electrode system is shown by the *R*_ct_ [39–40]. The straight line in the low-frequency range of the EIS graphic describes the capacitive properties [37]. The equivalent series resistance (*R*_ESR_), which is often generated from the ohmic and interfacial resistance of the electrolyte and electrode, respectively, is designated by the intersection of the semicircle on the real axis [37,41]. The minimal interfacial resistance is confirmed by the PG-CDs-AgNPs reduced *R*_ESR_ value. Easy electrolyte ion transport through highly conducting CDs is the primary cause of the decreased *R*_ESR_ and *R*_ct_ for PG-CDs-AgNPs. Above all, the fact that the straight line in the case of PG-CDs-AgNPs has a greater slope suggests that these particles have optimal supercapacitive properties and electrochemical activity. In the three-electrode investigation, PG-CDs-AgNPs showed better electrochemical activity overall in terms of capacitive performance, charge-transfer activity, and discharge time.

#### Electrochemical analysis of the asymmetric supercapacitor device in a 1 M TEABF_4_/DMSO organic electrolyte

**Figure 6:**
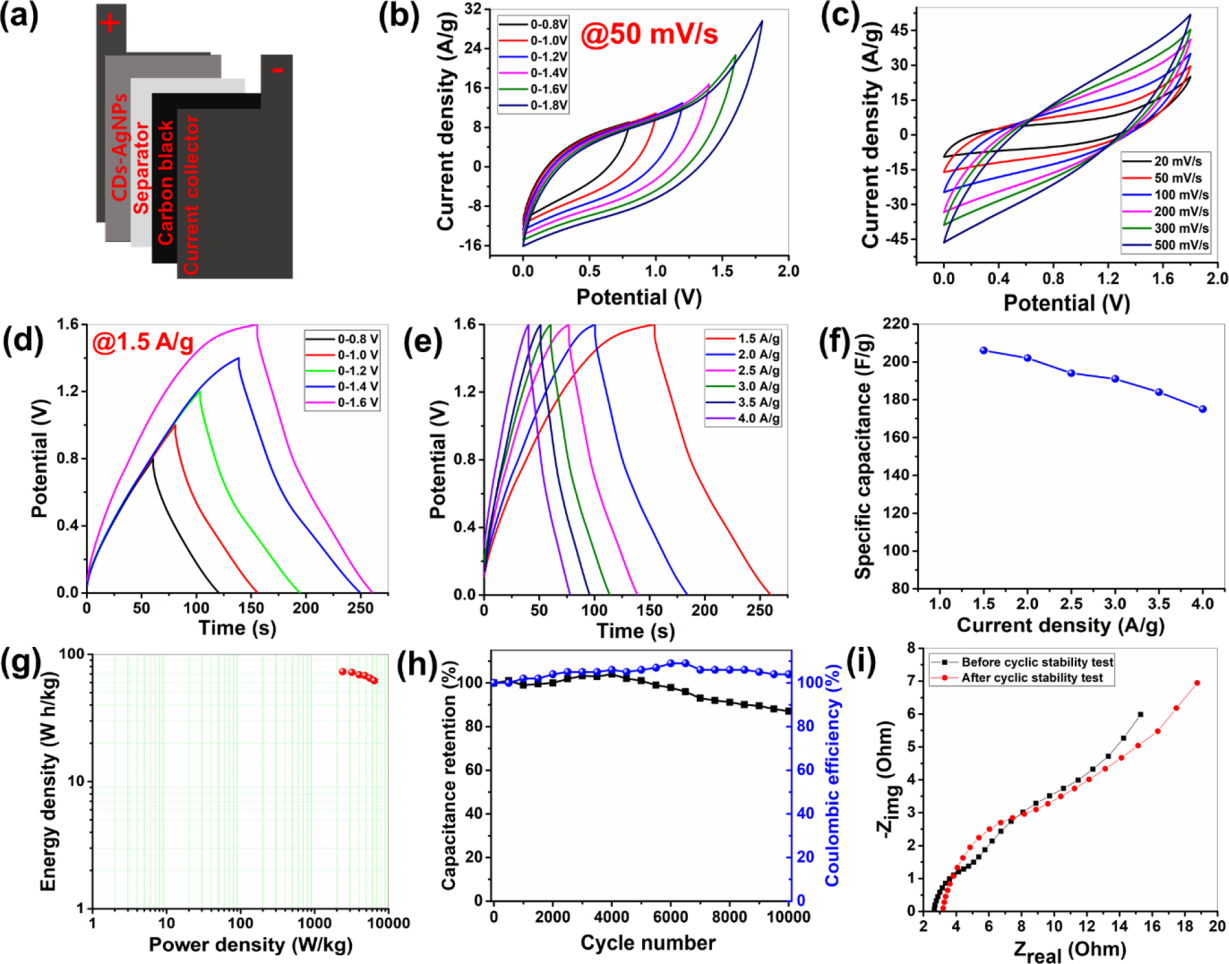
(a) Schematic image of the ASC device using PG-CDs-AgNPs and CB as positive and negative electrodes, respectively. In the organic electrolyte, the electrochemical evaluations of the ASC device are: (b) CV analysis in different voltage limits at 50 mV/s, (c) CV analysis at different scan rates (20–500 mV/s) within 0–1.8 V, (d) GCD plots in various potential limits at 1.5 A/g, (e) GCD profiles at various current densities (1.5–4 A/g) within 0–1.6 V, (f) SC as a function of current density graph of the ASC device, (g) Ragone plot, (h) cyclic stability and coulombic efficiency plot, and (i) before and after cyclic stability Nyquist plots.

Electrochemical evaluations attributed that in the three-electrode configuration, the PG-CDs-AgNPs electrode possessed superior electrochemical activity. Thus, utilizing carbon black (CB) as the negative electrode and PG-CDs-AgNPs as the positive electrode, an ASC device was built for real-time application. Supporting Information File 1, section S2 covers the device assembly processes. A figure of the ASC device is portrayed in Figure 6a. All electrochemical activities of the supercapacitor device were evaluated using 1 M TEABF_4_/DMSO, an organic electrolyte. The consistent operating potential window of the electrolyte is crucial for an electrochemical device. The overpotential of water limits the stable functioning voltages of aqueous electrolytes to about 1.0 V, despite the fact that they are readily available, easy to handle, and environmentally friendly. Organic electrolytes, on the other hand, provide larger potential windows for electrochemical devices. The organic electrolyte 1 M TEABF_4_/DMSO can provide a stable working potential window. Due to its strong polarity and decent ionic mobility, TEABF4/DMSO may be helpful in maximizing the electrochemical activity.

Comprehensive electrochemical experiments, such as cyclic stability, EIS, GCD, and CV evaluations, were conducted on the ASC device. A CV test (Figure 6b) of the ASC device was first performed at a fixed scan rate of 50 mV/s with changing the voltage windows (up to 1.8 V). This showed that the features of the CV curve did not expressively deviate in the organic electrolyte when the potential limits were varied from 1.0 to 1.8 V. The CV experiment shows that the ASC was functioning within the range of 0 to 1.8 V, which is the maximum stable operating potential. The CV profiles of the device are shown in Figure 6c at various scan rates (20–500 mV/s) within the 0–1.8 V maximum voltage limit. The areas and current responses of the cyclic voltammograms rose with an increasing scan rate, similar to the three-electrode investigation, due to the faster diffusion kinetics on the surface of the electrode. At high scan rates (such as 500 mV/s), there was no discernible diversion of the features of the CV profiles. The current responsiveness of the device was adequate even at a 20 mV/s scan rate. For real-time applications, the capacitive effectiveness of PG-CDs-AgNPs as an ASC electrode shows enormous promise. The largest pseudocapacitance contribution of the PG-CDs-AgNPs electrode was indicated by the CV graphs, which considerably diverged from their true rectangular shape. In order to determine the SC of the device, a GCD analysis was also carried out at various potential limitations (1.0–1.6 V); Figure 6d shows the GCD plots. The operating voltage limit was accompanied by an improvement in both the energy density (ED) and SC. Additionally, when the current density increased, the GCD plot features did not change (Figure 6e). Figure 6f demonstrates the current density as a function of SC of the ASC device at multiple current densities. The ASC reached the maximum SC of 200 F/g, while the maximum ED was 71 W·h/kg at 1.5 A/g in the organic electrolyte. The SC of 175 F/g was demonstrated by the ASC device even at a high current density of 4 A/g, confirming the required rate capacity of the PG-CDs-AgNPs-based ASC. Only around 12.5% of the initial SC was lost by the PG-CDs-AgNPs at a high current density. A suitable rate capability and decent electrochemical activities are ascribed to the contribution from pseudocapacitance and EDLC and the synergistic involvement of CDs and Ag NPs. The small IR drop seen by the ASC device indicates that all of the electrode capacitance is present within the operational voltage. Figure 6g illustrates the Ragone plot of the device which achieved the maximum ED of 71 W·h/kg. The ASC device also showed the highest power density (PD) of 6400 W/kg, while sustaining 62 W·h/kg ED. The stability of this ASC device over 10,000 GCD cycles was also assessed. After the accomplishment of 10,000 GCD cycles, the ASC showed ≈87% SC retention (Figure 6h). The capacitance retention of the device improved over the first 4000 cycles as the electrolyte ions gradually permeated the electrode pores, activating both the anode and cathode materials. A slight decline in the capacitance retention was primarily attributed to the progressive mechanical deformation of the electrode material and the partial detachment of active materials from the current collectors. The cyclic stability of the PG-CDs-AgNPs nanohybrid as the positive electrode for ASC has also been compared with similar types of electrodes, as summarized in Supporting Information File 1, Table S1. The Coulombic efficiency of the device was also measured up to 10000 cycles and the device exhibited almost 100% of coulombic efficiency. Following the cyclic stability test, the EIS analysis was also performed. The Nyquist plots of the device before and after stability tests are presented in Figure 6i. Before the cyclic stability test, the *R*_ESR_ value of this ASC was found to be 2.6 Ω. However, the *R*_ESR_ value was increased to 3.2 Ω after the stability test, and this is mostly owing to the deformation and degradation of the electrode material during the electrochemical progression.

## Conclusion

A green synthetic method was successfully adopted for the production of AgNPs using CDs produced from waste biomass as a stabilizing and reducing agent. The TEM analysis showed that the produced PG-CDs-AgNPs have more uniform surfaces and spherical shapes with an average particle size of 10–11 nm. The synthesized PG-CD-AgNPs are crystalline with CDs support, confirmed by SEAD patterns and XRD. The synthesized PG-CDs-AgNPs electrode showed a maximum SC value of 540 F/g at 1 A/g in the three-electrode system. Besides, the ASC device constructed with PG-CDs-AgNPs reached a decent energy density of 71 W·h/kg with ≈87% retention of SC after 10,000 GCD cycles. Decent electrochemical properties of PG-CDs-AgNPs confirm that PG-CDs-AgNPs could be a promising contender for energy storage devices.

## Supporting Information

File 1 Experimental procedures including materials details and characterizations; Schematic representation; BET isotherm and pore size distribution plot; SEM, EDX, and elemental mapping of PG-CDs-AgNPs; ASC device fabrication method; Table for the comparison of cyclic stability.

## Data Availability

Data generated and analyzed during this study is available from the corresponding author upon reasonable request.
