# Supplementary material for: Synthesis of biowaste-derived carbon-dot-mediated silver nanoparticles and the evaluation of electrochemical properties for supercapacitor electrodes
Source: Beilstein J Nanotechnol. 2025 Jun 24;16:933–43. doi: 10.3762/bjnano.16.71 (PMC12207810; doi:10.3762/bjnano.16.71)
Supplement: File 1 — Experimental procedures including materials details and characterizations; Schematic representation; BET isotherm and pore size distribution plot; SEM, EDX, and elemental mapping of PG-CDs-AgNPs; ASC device fabrication method; Table for the comparison of cyclic stability. [file Beilstein_J_Nanotechnol-16-933-s001.pdf]

BEILSTEIN JOURNAL OF NANOTECHNOLOGY

Supporting Information

for

Synthesis of biowaste-derived carbon-dot-mediated silver nanoparticles and the evaluation of electrochemical properties for supercapacitor electrodes

Navya Kumari Tenkayala, Chandan Kumar Maity, Md Moniruzzaman and Subramani Devaraju

Beilstein J. Nanotechnol. **2025,** *16,* 933–943. doi:10.3762/bjnano.16.71

Experimental procedures including materials details and characterizations; Schematic representation; BET isotherm and pore size distribution plot; SEM, EDX, and elemental mapping of PG-CDs-AgNPs; ASC device fabrication method; Table for the comparison of cyclic stability

License and Terms: This is a supporting information file under the terms of the Creative Commons Attribution License (https://creativecommons.org/licenses/by/4.0). Please note that the reuse, redistribution and reproduction in particular requires that the author(s) and source are credited and that individual graphics may be subject to special legal provisions.
The license is subject to the *Beilstein Journal of Nanotechnology* terms and conditions: (https://www.beilstein-journals.org/bjnano/terms)

S1. Experimental section

Materials

All the chemicals were used without further purification. Silver nitrate ($AgNO_3$) was purchased from Sisco Research Laboratories, India. Dimethyl sulfoxide (DMSO, 99.5%), potassium chloride (KCl), tetraethylammonium tetrafluoroborate ($TEABF_4$, 99%), and ethanol were purchased from Sigma-Aldrich, India. Deionized (DI) water was used for all syntheses and experiments.

Preparation of *Pongamia pinnata* leaves powder

Waste *Pongamia pinnata* leaves were collected from nearby places, washed with distilled water, and dried in the hot air woven at 70 °C. Then, using a mortar and pestle, the dried leaves were ground into fine powder and used for further processing.

Material characterization

Using a Shimadzu UV 1800 spectrophotometer, the UV–visible absorption spectra of PG-CDs and PG-CDs-AgNPs were acquired. The PL emission spectra of PG-CDs and PG-CDs-AgNPs were taken using a spectrophotometer (RF 6000, Shimadzu, Japan). X-ray diffraction patterns of PG-CDs-AgNPs were measured using a powder X-ray diffractometer (Model-D8 Advance Bruker AXS powder XRD). At a degassing temperature of 60 °C for 4 h, BET analysis and pore size distribution of the samples were measured via N_2 adsorption–desorption method using the Micromeritics' ASAP 200. A Thermo Fisher Scientific-MultiLab-2000, (monochromated Al Kα source) was used for the XPS analysis. A Hitachi's scanning electron microscope (SEM) (SU8600) was utilized to visualize the surface morphologies (operating

voltage: 5kV) and elemental mapping of Ag, C, and O. By using a JEOL JEM-ARM200F (operating voltage: 200kV), transmission electron microscopy (TEM) analysis was performed.

Electrochemical characterization

For three-electrode analysis, cyclic voltammetry (CV), galvanostatic charging/discharging (GCD), and electrochemical impedance spectroscopy (EIS) were performed using 1 M aqueous (aq.) KCl as the electrolyte on a Biologic SP200 electrochemical workstation. Pt foil and Ag/AgCl were utilized as the counter and reference electrodes in the three-electrode configuration, while the electroactive materials (PG-CDs-AgNPs) [sample: carbon black: binder (Nafion) = 80:15: 5] were mounted on the flat surface of a graphite rod to fabricate the working electrode. For GCD and CV analyses in the aqueous electrolyte, a potential limit of 0.0–0.8 V was maintained to prevent the overpotential of H_2O. The EIS study was carried out between 1 MHz and 1 Hz at room temperature. The gravimetric specific capacitance (SC) of PG-CDs-AgNPs was calculated applying the following equation [1]:

$$SC\ (F/g) = \frac{i \times t}{m \times \Delta V} \qquad (S1)$$

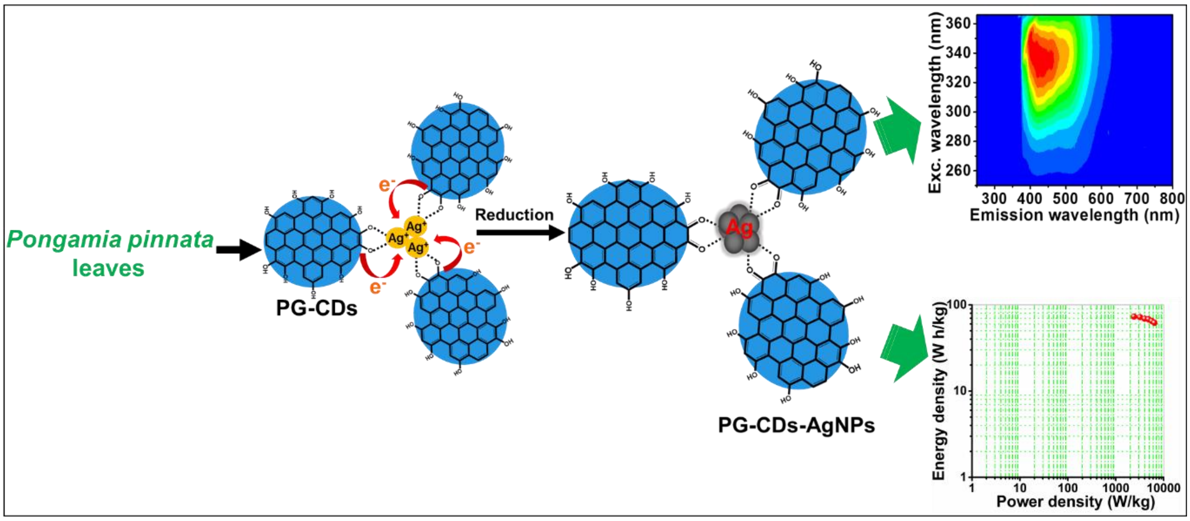

Scheme S1: Schematic representation of the overall methodology as well as the optical and electrochemical storage properties of PG-CDs-AgNPs.

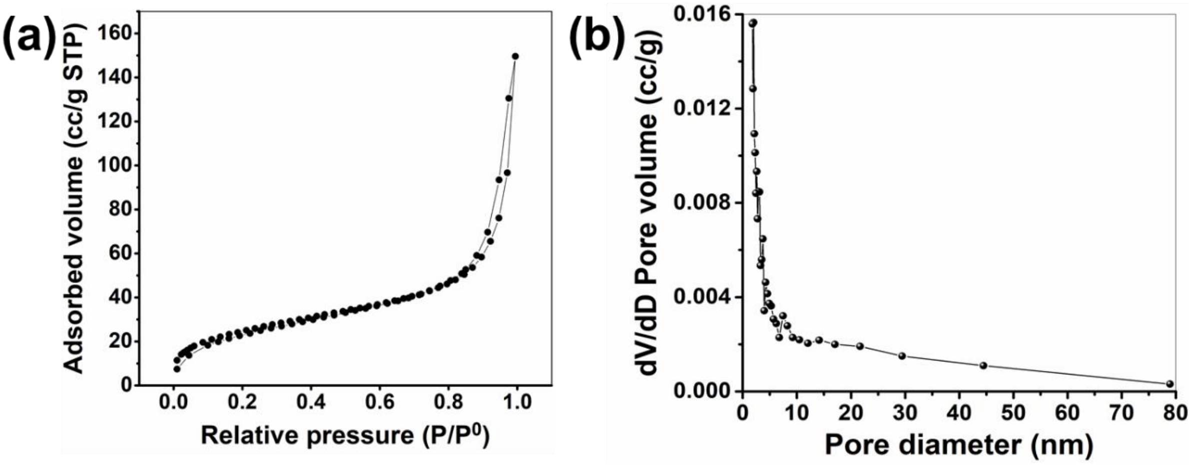

Figure S1: (a) BET isotherm and (b) pore size distribution plot of PG-CDs-AgNPs.

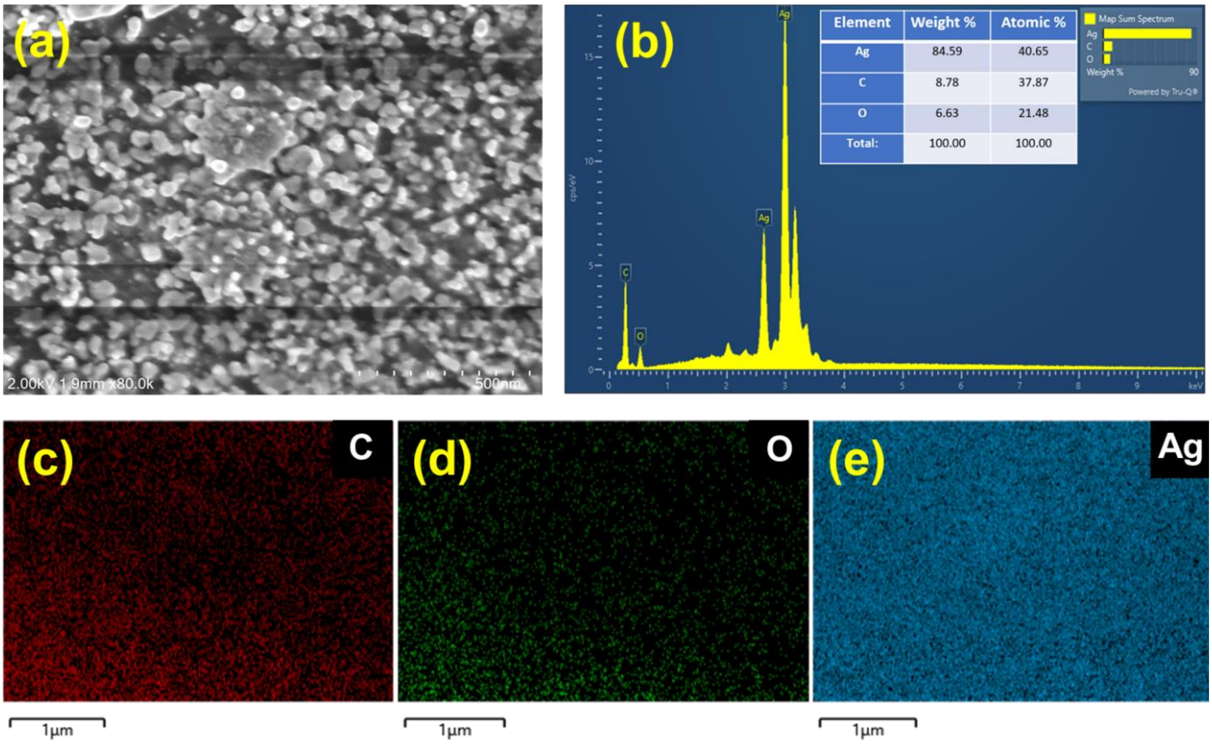

Figure S2: (a) SEM image of PG-CD-AgNPs, (b) EDX analysis of PG-CD-AgNPs, and (c–e) Elemental mapping of C, O, and Ag, respectively for PG-CD-AgNPs.

S2. Asymmetric supercapacitor device fabrication method

For the fabrication of an asymmetric supercapacitor (ASC) device, PG-CD-AgNPs and carbon black (CB) were used as positive and negative electrodes, respectively; whereas tissue paper acted as the separator. In a manner similar to [2], the ASC device was fabricated. The electrochemical analyses of the supercapacitor device were conducted using an organic electrolyte [1M tetraethylammonium tetrafluoroborate ($TEABF_4$)/ dimethyl sulfoxide (DMSO)] on a Biologic SP200 electrochemical workstation.

The gravimetric specific capacitance (SC) of the device was calculated applying the following equation [1]:

$$\mathrm{SC\ (F/g)} = \frac{2 \times i \times \Delta t}{m \times \Delta V} \qquad \text{(S2)}$$

The specific energy density (ED) and power density (PD) of the ASC device were calculated using the following equation [1]:

$$\mathrm{ED\ (W\ h/kg)} = \frac{SC \times \Delta V^2}{7.2} \qquad \text{(S3)}$$

$$\mathrm{PD\ (W/kg)} = \frac{ED \times 3600}{\Delta t} \qquad \text{(S4)}$$

Table S1: Comparison of the cyclic stability of the PG-CDs-AgNPs nanohybrid (as the positive electrode used in ASC) with other similar electrodes.

Electrode	Measurement type	Cyclic stability	Reference
Sn/SnO_2/GQD/CNF	As positive electrode in ASC	86.1% After 5000 cycles	[3]
Graphene oxide/CDs/ Polypyrrole	Symmetric supercapacitor	92.9% After 5000 cycles	[4]
AgNPs/Porous carbon	As positive electrode in ASC	82.7% After 10000 cycles	[5]
NF/NiSe/MnO_2-LCDs	As positive electrode in ASC	88.46% After 7000 cycles	[6]
Ag QDs/$NiMoO_4$	As positive electrode in ASC	84.4% After 5000 cycles	[7]
Ag quantum dots/MoO_3	As positive electrode in ASC	90% After 5000 cycles	[8]
Pt-AgNPs/rGO	Symmetric supercapacitor	70.65% After 10000 cycles	[9]
Ag@Ti_3C_2	As anode in ASC	91.27% After 5000 cycles	[10]
Zn–MoS_2/CDs@BN	As positive electrode in ASC	89% After 10000 cycles	[11]
NiCo-LDH@CDs	As positive electrode in ASC	78.6% After 10000 cycles	[12]
CDs/Ni-Zn phosphates	As positive electrode in ASC	90.6% After 10000 cycles	[13]
**PG-CDs-AgNPs**	As positive electrode in ASC	87% After 10000 cycles	This work

GQD: graphene quantum dot, CNF: carbon nanofiber, NF: nickel foam, LCDs: lignin-derived carbon dots, rGO: reduced graphene oxide, BN: boron nitride, and LDH: layered double hydroxide.

References

1. Chuai, M.; Zhang, K.; Chen, X.; Tong, Y.; Zhang, H.; Zhang, M. *Chem. Eng. J.* **2020,** *381,* 122682. doi:10.1016/j.cej.2019.122682.
2. Maity, C. K.; De, S.; Acharya, S.; Siddiki, S. H.; Sahoo, S.; Verma, K.; Thakur, V. K.; Nayak, G. C. *J. Energy Storage* **2022,** *56,* 105928. doi:10.1016/j.est.2022.105928.
3. Li, Z.; Zhang, C.; Bu, J.; Zhang, L.; Cheng, L.; Wu, M. *Carbon* **2022,** *194,* 197–206. doi:10.1016/j.carbon.2022.03.079.
4. Zhang, X.; Wang, J.; Liu, J.; Wu, J.; Chen, H.; Bi, H. *Carbon* **2017,** *115,* 134–146. doi:10.1016/j.carbon.2017.01.005.
5. Wang, R.; Li, X.; Nie, Z.; Jing, Q.; Zhao, Y.; Song, H.; Wang, H. *J. Energy Storage,* **2022,** *51,* 104364. doi:10.1016/j.est.2022.104364.
6. Xie, X.; Xu, Y.; Liu, J.; Wang, D. Lv, T.; Yuan, F.; Zhang, Q. *ACS Appl. Mater. Interfaces* **2024,** *16,* 68157-68168. doi:10.1021/acsami.4c13282.
7. Zhang, X.; Li, Z.; Yu, Z.; Wei, L.; Guo, X. *Appl. Surf. Sci.* **2020,** *505,* 144513. doi:10.1016/j.apsusc.2019.144513.
8. Zhang, X.; Fu, Q.; Huang, H.; Wei, L.; Guo, X. *Small,* **2019,** *15,* 1805235. doi:10.1002/smll.201805235.
9. Punnakkal, N.; Chandhana, J. P.; Nair, D.; Thomas, S. S.; Madhavan, A.; TG, S. B.; Vasu, S. P. *J. Power Sources* **2025,** *641,* 236819. doi:10.1016/j.jpowsour.2025.236819.
10. Alam, A.; Saeed, G.; Kim, K. W.; Kim, J. K.; Park, H. S.; Lim, S. *J. Energy Storage* **2023,** *72,* 108227. doi:10.1016/j.est.2023.108227.
11. Maity, C. K.; De, S.; Panigrahi, A.; Acharya, S.; Verma, K.; Kim, M. J.; Nayak, G. C. *Compos. B: Eng.* **2023,** *264,* 110887. doi:10.1016/j.compositesb.2023.110887.
12. Wang, S.; Deng, J.; Li, M.; Lin, J.; Luo, L.; Yuan, Z.; Zhang, W.; He, C.; Du, G.; Zhao, W. *Chem. Eng. J.* **2025,** *511,* 162275. doi:10.1016/j.compositesb.2023.110887.
13. Guo, M.; Wang, S.; Zhao, L.; Guo, Z. *Electrochim. Acta* **2018,** *292,* 299-308. doi:10.1016/j.electacta.2018.08.119.
